# Loneliness, immunological recovery patterns, and health-related quality of life (HRQOL) outcomes in patients receiving hematopoietic stem cell transplantation

**DOI:** 10.1186/s40359-024-01535-w

**Published:** 2024-01-19

**Authors:** Lori J. Lange, Steven C. Ames, Gretchen E. Ames, Michael G. Heckman, Launia J. White, Vivek Roy, James M. Foran

**Affiliations:** 1 Department of Psychology, University of North Florida, 1 UNF Drive, 32224 Jacksonville, FL USA; 2 Division of Hematology and Oncology, Mayo Clinic Florida, 4500 San Pablo Road South, 32224 Jacksonville, FL USA; 3 Department of Surgery, Mayo Clinic Florida, 4500 San Pablo Road South, 32224 Jacksonville, FL USA; 4 Division of Clinical Trials and Biostatistics, Mayo Clinic Florida, 4500 San Pablo Road South, 32224 Jacksonville, FL USA

**Keywords:** Loneliness, Hematopoietic stem cell transplant, Health-related quality of life (HRQOL), Psychoneuroimmunology, Psychosocial oncology

## Abstract

**Purpose:**

Loneliness may compromise health-related quality of life (HRQOL) outcomes and the immunological impacts of loneliness via neuroendocrinological mechanisms likely have consequences for patients who have undergone a hematopoietic stem cell transplantation (HSCT).

**Research approach and measures:**

Loneliness (pre-transplant), immunological recovery (Day 30, Day 100, 1-year post-transplant), and HRQOL (Day 100, 1 year) were measured in a sample of 205 patients completing a HSCT (127 autologous, 78 allogenic).

**Results:**

Greater levels of pre-transplant loneliness predicted poorer HRQOL at Day 100 and 1-year follow-up. Loneliness also was associated with higher absolute neutrophil to absolute lymphocyte (ANC/ALC) ratios in the entire sample at Day 30, which in turn was associated with Day 100 HRQOL.

**Conclusions:**

Findings demonstrate that pretransplant loneliness predicts HRQOL outcomes and associates with inflammatory immunological recovery patterns in HSCT patients. The balance of innate neutrophils to adaptive lymphocytes at Day 30 present a distinct profile in lonely individuals, with this immunity recovery profile predicting reduced HRQOL 100 days after the transplant. Addressing perceptions of loneliness before HSCT may be an important factor in improving immunological recovery and HRQOL outcomes.

**Supplementary Information:**

The online version contains supplementary material available at 10.1186/s40359-024-01535-w.

## Introduction

A hematopoietic stem cell transplantation (HSCT) is an arduous medical procedure which carries a high risk of mortality and morbidity post-transplant. Chemotherapy, sometimes with radiation, is used to eradicate disease cells prior to an autologous (i.e., hematopoietic stem cells from self) or allogeneic (i.e., hematopoietic stem cells or bone marrow from a matched donor) transplant from which patients rebuild their immune system. HSCT often requires 3–4 weeks of hospitalization and may involve extended periods of isolation and recovery, with demands particularly distressing compared to other cancer treatments [1, 2]. High levels of emotional distress have been observed in patients during hospitalization, especially during the transplant anticipatory period [2–4]. Social and emotional quality of life, among other health-related quality of life (HRQOL) indicators, are compromised during in-patient hospitalization with improvements progressing over the months following the transplant [5–7]. However, variability exists in post-transplant HRQOL and recovery, and the use of theoretically-driven work using established conceptual measures is important for determining social, psychological, biological, and other contributors to HSCT outcomes.

A myriad of psychosocial factors have been found to be predictive of health outcomes in oncology populations [e.g., 8, 9, 10, 11]. Comparatively few studies have investigated psychosocial predictors of HSCT outcomes, and some inconsistencies in findings may result from methodological limitations [1, 12, 13]. Loneliness has received considerable attention in the health literature, as large-scale longitudinal studies have established it as a risk factor for pro-inflammatory illness morbidity and all-cause mortality [14–17]. Because the subjective aspects of social connections appear to be more important than objective realities of social network characteristics in determining physical and psychological well-being, loneliness is defined as distress due to *perceived* isolation and deficiencies in the desired quantity and quality of relationships [18–20].

According to the Loneliness Model [18, 21, 22], human beings have a basic need to feel connected to others, with unsafe feelings arising when this need is unmet. Perceived inadequacies in social connections leads to threat hypervigilance and stress with cognitive-perceptual biases that perpetuate social isolation. One of the mechanisms by which loneliness can impact health is through stress and its direct impacts on neurobiological processes [18, 23, 24]. Of particular significance to the HSCT population is the neuroendocrinological effects of loneliness on the immune system. In fact, Costanzo and colleagues [12] state the widely-accepted view that “many of the host- or recipient-derived cells essential to the recovery of hematopoiesis and immunity also express receptors for factors that are responsive to the extensive crosstalk between psychological state and the neuroendocrine and immune systems” (p. 5).

The stress of loneliness causes a chain of physiological responses by which the autonomic nervous system and hypothalamic-pituitary adrenocortical (HPA) axis release stress hormones, such as catecholamines and glucocorticoids [25–28]. Immunological and regulatory cells contain adrenergic and/or glucocorticoid receptors, thus providing a mechanism by which psychosocial factors, such as loneliness, impact humoral and cell-mediated immune recovery and functioning. A well-established effect of glucocorticoids involves the regulatory distribution of circulating leukocytes in the blood. Of relevance to the current study, cortisol acts on glucocorticoid receptors to increase circulating neutrophils (neutrophilia) and decrease circulating lymphocytes (lymphopenia) and monocytes (monocytopenia) [29–31]. Accordingly, higher neutrophil/lymphocyte and neutrophil/monocyte hematological ratios are associated with elevated cortisol levels [29].

**Fig. 1:**
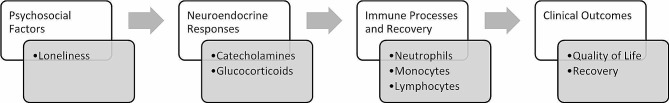
Biobehavioral model pathways by which loneliness contributes to HSCT recovery and outcomes. Figure adapted from Costanzo et al. [12] and Knight et al. [1], with variables of interest for the current study identified in shaded boxes

Loneliness has been found to be associated with increased distress in HSCT survivors [32] and a study of long-term survivors found that 70% of those reporting elevated distress also reported feeling lonely [33]. The psychosocial biobehavioral mechanisms outlined by Costanzo and colleagues [12], and elaborated on by Knight and colleagues [1], reveal convincing pathways by which psychosocial factors may impact HSCT immunological recovery and outcomes (See Fig. 1 for a model adapted for the current study).

The current study seeks to investigate HRQOL and immunological impacts of experienced loneliness during hospitalization from a HSCT. More research is needed to investigate psychosocial factors and pathways in this population. A few studies have investigated psychosocial factors and immunological recovery in HSCT, finding optimism and reduced anxiety associated with faster neutrophil engraftment [34] and pretreatment distress (anxiety, depression) predicting slower white blood cell count recovery post-transplant [35]. No known study has investigated the association of loneliness with immunological recovery from an autologous transplant. The current study takes a unique approach by investigating loneliness at baseline as a predictor of immunological parameters in HSCT patients at 30 days, 100 days, and 1 year post-transplant and HRQOL outcomes (Day 100, 1 year). Loneliness (pre-transplant) and immunological markers (Day 30, Day 100, 1 year) also are associated with HRQOL outcomes (Day 100, 1 year). Based upon theoretical and empirical findings, we predict that the stress of loneliness will predict immunological recovery such that patterns will show enhanced neutrophil and suppressed monocyte and lymphocyte levels through measures of absolute counts and ratios. Furthermore, it is hypothesized that immunological recovery patterns associated with loneliness will predict poorer HRQOL outcomes at 100 days and 1 year following HSCT.

## Methodology

### Participants

The current study included a total of 205 patients (autologous *n* = 127; allogenic *n* = 78) out of 662 patients who underwent a hematopoietic stem cell transplantation (HSCT) at the Mayo Clinic in Jacksonville between December 2014 and October 2020. This study was approved by Institutional Review Boards at the University of North Florida and Mayo Clinic at Jacksonville (IRB# 14-004628). All consenting participants were over 18 years of age and were informed that they could discontinue study participation at any time without it affecting their care at the Mayo Clinic or any other medical facility.

Demographic and disease and treatment information were accessible from patient medical. To stay consistent with recommended research integrity recommendations for controlling for transplant heterogeneity [1, 12, 13], autologous and allogenic transplant groups were analyzed as distinct groups in addition to analyses on the sample as a whole. Refer to Table 1 for sample characteristics measured pre-transplant at baseline. Patient outcomes are summarized in Table 2.

**Table 1:** Patient baseline characteristics

	Autologous transplant (*N* = 127)		Allogeneic transplant (*N* = 78)		All patients (*N* = 205)	
Variable	N	Median (minimum, maximum) or No. (%) of patients	N	Median (minimum, maximum) or No. (%) of patients	N	Median (minimum, maximum) or No. (%) of patients
Age at transplant (years)	127	60 (22, 80)	78	59 (22, 74)	205	60 (22, 80)
Sex (Male)	127	68 (53.5%)	78	39 (50.0%)	205	107 (52.2%)
Ethnicity (Hispanic/Latino)	126	6 (4.8%)	78	5 (6.4%)	204	11 (5.4%)
Race (non-White)	125	21 (16.8%)	78	5 (6.4%)	204	26 (12.8%)
Marital status	127		78		205	
Never married		7 (5.5%)		5 (6.4%)		12 (5.9%)
Currently married		96 (75.6%)		55 (70.5%)		151 (73.7%)
Separated		3 (2.4%)		1 (1.3%)		4 (2.0%)
Divorced		15 (11.8%)		8 (10.3%)		23 (11.2%)
Widowed		2 (1.6%)		5 (6.4%)		7 (3.4%)
Cohabitating with significant other		4 (3.1%)		4 (5.1%)		8 (3.9%)
Level of school completed	127		77		204	
Some HS or less		7 (5.5%)		5 (6.5%)		12 (5.9%)
High school grad/GED		28 (22.0%)		15 (19.5%)		43 (21.1%)
Some college, associate’s or technical/VOC school		32 (25.2%)		26 (33.8%)		58 (28.4%)
College graduate		37 (29.1%)		24 (31.2%)		61 (29.9%)
Graduate school		23 (18.1%)		7 (9.1%)		30 (14.7%)
Current employment status	127		78		205	
Full-time		38 (29.9%)		29 (37.2%)		67 (32.7%)
Part-time		7 (5.5%)		6 (7.7%)		13 (6.3%)
On leave with pay		12 (9.4%)		10 (12.8%)		22 (10.7%)
On leave without pay		4 (3.1%)		6 (7.7%)		10 (4.9%)
Disabled		20 (15.7%)		5 (6.4%)		25 (12.2%)
Unemployed		3 (2.4%)		2 (2.6%)		5 (2.4%)
Retired		39 (30.7%)		16 (20.5%)		55 (26.8%)
Homemaker		3 (2.4%)		2 (2.6%)		5 (2.4%)
Student		1 (0.8%)		2 (2.6%)		3 (1.5%)
Approximate annual household gross income	122		76		198	
Less than $20,000		9 (7.4%)		5 (6.6%)		14 (7.1%)
$20,000-$39,999		19 (15.6%)		9 (11.8%)		28 (14.1%)
$40,000-$59,999		18 (14.8%)		11 (14.5%)		29 (14.6%)
$60,000-$79,999		22 (18.0%)		17 (22.4%)		39 (19.7%)
$80,000-$99,999		22 (18.0%)		12 (15.8%)		34 (17.2%)
$100,000 or more		32 (26.2%)		22 (28.9%)		54 (27.3%)
Smoking history	127		77		204	
Never smoked		69 (54.3%)		42 (54.5%)		111 (54.4%)
Past smoker		53 (41.7%)		31 (40.3%)		84 (41.2%)
Current smoker		5 (3.9%)		4 (5.2%)		9 (4.4%)
Illicit drug use						
Any illicit drug use	127	7 (5.5%)	78	6 (7.7%)	205	13 (6.3%)
Marijuana	127	7 (5.5%)	78	5 (6.4%)	205	12 (5.9%)
Prescription drugs	127	0 (0.0%)	78	1 (1.3%)	205	1 (0.5%)
Primary disease type	127		78		205	
ALL		0 (0.0%)		12 (15.4%)		12 (5.9%)
AML		0 (0.0%)		25 (32.1%)		25 (12.2%)
Other acute leukemia		0 (0.0%)		2 (2.6%)		2 (1.0%)
CML		0 (0.0%)		5 (6.4%)		5 (2.4%)
MDS/MPD		0 (0.0%)		22 (28.2%)		22 (10.7%)
Hodgkin’s disease		8 (6.3%)		4 (5.1%)		12 (5.9%)
NHL		27 (21.3%)		3 (3.8%)		30 (14.6%)
PCD		89 (70.1%)		1 (1.3%)		90 (43.9%)
Aplastic anemia		0 (0.0%)		1 (1.3%)		1 (0.5%)
CLL		1 (0.8%)		1 (1.3%)		2 (1.0%)
Other		2 (1.6%)		2 (2.6%)		4 (2.0%)
Type of induction chemotherapy	127		77		204	
BuCy		0 (0.0%)		11 (14.3%)		11 (5.4%)
CyTBI		0 (0.0%)		5 (6.5%)		5 (2.5%)
FluBu		0 (0.0%)		49 (63.6%)		49 (24.0%)
FluMel		0 (0.0%)		3 (3.9%)		3 (1.5%)
Mel		90 (70.9%)		0 (0.0%)		90 (44.1%)
BEAM		37 (29.1%)		1 (1.3%)		38 (18.6%)
FluCyTBI		0 (0.0%)		4 (5.2%)		4 (2.0%)
Other		0 (0.0%)		4 (5.2%)		4 (2.0%)
How often do you have a drink containing alcohol?	126		78		204	
Never		62 (49.2%)		34 (43.6%)		96 (47.1%)
Monthly or less		34 (27.0%)		24 (30.8%)		58 (28.4%)
2 to 4 times a month		14 (11.1%)		14 (17.9%)		28 (13.7%)
2 to 3 times a week		9 (7.1%)		4 (5.1%)		13 (6.4%)
4 or more times a week		7 (5.6%)		2 (2.6%)		9 (4.4%)
BMI	123	28.8 (0.0, 55.6)	77	28.1 (18.9, 47.0)	200	28.5 (0.0, 55.6)
ANC	115	3.3 (0.6, 68.9)	68	2.1 (0.1, 35.8)	183	2.8 (0.1, 68.9)
ALC	115	1.0 (0.1, 5.6)	68	0.9 (0.1, 6.5)	183	0.9 (0.1, 6.5)
AMC	115	0.6 (0.1, 4.7)	68	0.4 (0.0, 2.5)	183	0.5 (0.0, 4.7)
ANC/AMC	115	5.7 (1.4, 144.0)	68	6.0 (0.6, 88.3)	183	5.8 (0.6, 144.0)
ANC/ALC	115	3.4 (0.8, 175.8)	68	2.5 (0.1, 27.8)	183	3.0 (0.1, 175.8)
UCLA loneliness total score	116	26 (20, 61)	67	30 (20, 57)	183	28 (20, 61)

**Table 2:** Patient outcomes

	Autologous transplant (*N* = 127)		Allogeneic transplant (*N* = 78)		All patients (*N* = 205)	
Variable	N	Median (minimum, maximum) or No. (%) of patients	N	Median (minimum, maximum) or No. (%) of patients	N	Median (minimum, maximum) or No. (%) of patients
FACT-BMT total score						
Day 100	104	120.5 (79.6, 146.1)	52	110.6 (42.0, 136.0)	156	117.0 (42.0, 146.1)
1 year	97	122.0 (57.1, 147.0)	44	118.1 (42.0, 147.0)	141	121.1 (42.0, 147.0)
Days hospitalized during transplant	122	16.5 (2.0, 36.0)	70	25.5 (12.0, 53.0)	192	19.0 (2.0, 53.0)
Total days hospitalized for re-admission	121	0.0 (0.0, 55.0)	70	0.0 (0.0, 115.0)	191	0.0 (0.0, 115.0)
Graft vs. host disease	120	1 (0.8%)	70	34 (48.6%)	190	35 (18.4%)
ANC at day 30	115	2.3 (0.7, 16.4)	71	2.9 (0.7, 11.0)	186	2.5 (0.7, 16.4)
ALC at day 30	115	1.2 (0.4, 5.3))	71	0.7 (0.0, 1.8)	186	1.0 (0.0, 5.3)
AMC at day 30	115	0.8 (0.1, 2.9)	71	0.9 (0.0, 2.4)	186	0.8 (0.0, 2.9)
ANC/AMC at day 30	115	3.0 (0.3, 34.1)	71	3.8 (0.6, 170.5)	186	3.2 (0.3, 170.5)
ANC/ALC at day 30	115	2.0 (0.4, 16.9)	71	4.9 (1.1, 141.0)	186	2.5 (0.4, 141.0)
ANC at day 100	102	2.7 (0.3, 9.9)	58	2.7 (0.3, 15.2)	160	2.7 (0.3, 15.2)
ALC at day 100	102	1.1 (0.3, 3.1)	58	0.8 (0.1, 9.6)	160	1.0 (0.1, 9.6)
AMC at day 100	102	0.5 (0.1, 1.5)	58	0.6 (0.0, 5.9)	160	0.5 (0.0, 5.9)
ANC/AMC at day 100	102	5.6 (0.9, 45.7)	58	4.9 (0.8, 94.0)	160	5.4 (0.8, 94.0)
ANC/ALC at day 100	102	2.2 (0.1, 10.2)	58	3.0 (0.5, 23.9)	160	2.4 (0.1, 23.9)
ANC at 1 year	73	2.8 (0.8, 46.8)	44	3.4 (0.1, 12.3)	117	3.0 (0.1, 46.8)
ALC at 1 year	73	1.2 (0.3, 6.5)	44	1.3 (0.1, 4.1)	117	1.3 (0.1, 6.5)
AMC at 1 year	73	0.5 (0.2, 1.1)	44	0.7 (0.1, 1.5)	117	0.5 (0.1, 1.5)
ANC/AMC at 1 year	73	5.6 (2.4, 49.8)	44	5.3 (1.6, 17.6)	117	5.3 (1.6, 49.8)
ANC/ALC at 1 year	73	2.3 (0.6, 15.0)	44	2.8 (0.1, 17.7)	117	2.4 (0.1, 17.7)
Recurrence	113	20 (17.7%)	67	9 (13.4%)	180	29 (16.1%)
Death	120	4 (3.3%)	69	17 (24.6%)	189	21 (11.1%)

### Measures

White blood cell counts (i.e., absolute neutrophil count, absolute monocyte count, absolute lymphocyte count) were extracted from each patient’s medical file. Day 30 counts were obtained between day 23 to day 37 post-transplant, and Day 100 counts were obtained from the follow-up appointment with a physician 100 days and one year after the transplant. The neutrophil to lymphocyte ratio (ANC/ALC) was calculated by dividing absolute neutrophil count by absolute lymphocyte count. Absolute neutrophil counts were divided by absolute monocyte counts to derive the neutrophil to monocyte ratios (ANC/AMC).

Participants completed surveys at the following timepoints: (1) at the time of the pre-transplant team assessment, (2) during the medical evaluation at day 100, and (3) at the time of the first annual medical evaluation at 1 year. For patients with missing data for more than 50% of the individual questions for a given measure, the total score was considered to be missing. For patients with missing data for less than 50% of the individual questions for a given measure, the missing values were imputed using the average value of the patients who answered the given question.

#### UCLA loneliness scale version 3

The UCLA Loneliness Scale Version 3 [36] was used to assess general loneliness at baseline, pre-transplant only. Feelings of loneliness are rated on a Likert scale from 0 “*Never*” to 4 “*Always*”, with some items reversed scored so that higher scores on the UCLA Loneliness Scale indicate greater loneliness. The internal consistency for the UCLA Loneliness Scale Version 3 is reliable with Cronbach’s α ranging from 0.89 to 0.94 and a test-retest reliability of *r* = 0.73 [36]. UCLA loneliness total scores ranged from 20 to 61 with a median of 26 in the current sample.

#### Functional assessment of cancer therapy-bone marrow transplant (FACT-BMT) scale

The FACT-BMT [37] was measured at both 100 days and 1 year following HSCT, and is a validated, cancer specific quality of life instrument, measuring four well-being subscales (physical, social/family, emotional, and functional) and a bone marrow transplantation specific subscale (additional concerns). The questions are rated on a four-point Likert scale from 0 “*Not at all*” to 4 “*Very much*”. Certain items are reverse-scored so that higher summed scores denote better functioning (ranging from 0 to 148). Instructions on handling missing data and calculating subscale and summary scores were followed according to the recommendations of McQuellon et al. [37]. Reliability for the FACT-BMT scale and subscales ranged from Cronbach’s α’s of 0.86 to 0.89, with the BMT subscale ranging from 0.54 to 0.63 [37]. Overall HRQOL at 100 days (*M* = 113.9, *SD* = 20.30) in the current study was similar, only slightly higher, to that reported in a separate bone marrow transplant study [37] (*M* = 112.0, *SD* = 20.3).

### Data analyses

Comparisons of ANC, ALC, AMC, ANC/AMC, ANC/ALC, and FACT-BMT between different time points were made using paired t-tests. Associations between UCLA loneliness total score at baseline with outcomes (separately in the autologous transplant subgroup, the allogeneic transplant subgroup, and in all patients) were evaluated using multivariable regression models appropriate for the nature of the given outcome (continuous or count), where outcomes that were measured at multiple time points were assessed in separate regression models. Models were adjusted for the pre-defined potential confounding variables of age at transplant, sex, ethnicity, race, smoking history, current drinking, and BMI. Additionally, transplant subgroup was adjusted for in analyses of all patients. Regarding the specific statistical models utilized, linear regression models were used for continuous outcomes (FACT-BMT total score, ANC, ALC, AMC, ANC/AMC, ANC/ALC) and negative binomial regression models were used for count outcomes (days hospitalized during transplant). ANC, ALC, AMC, ANC/AMC, and ANC/ALC were all examined on the logarithm scale in all regression analyses owing to their skewed distributions.

For linear regression models, regression coefficients and 95% confidence intervals (CIs) were estimated and are interpreted as the increase in the mean outcome measure corresponding to a 5-unit increase in UCLA loneliness total score. For negative binomial regression models, multiplicative effects on the mean and 95% CIs were estimated and are interpreted as the multiplicative increase on the mean outcome measure corresponding to a specified increase in the given psychological factor. Additionally, associations of ANC, ALC, and AMC-related measures with UCLA loneliness total score at baseline and FACT-BMT total score at day 100 and 1 year were assessed using the aforementioned multivariable linear regression models; regression coefficients correspond to each doubling in the given ANC, ALC, or AMC-related measure.

We utilized a Bonferroni correction for multiple testing in order to account for the 7 general outcome measures that were examined for association with UCLA loneliness total score in the primary analysis, after which p-values < 0.0071 were considered as statistically significant. P-values < 0.05 were considered as statistically significant in all other secondary analyses. All statistical tests were two-sided. Statistical analyses were performed using SAS (version 9.4; SAS Institute, Inc., Cary, North Carolina) and R Statistical Software (version 4.1.2; R Foundation for Statistical Computing, Vienna, Austria).

## Results

Associations of UCLA loneliness total score at baseline with outcomes are displayed in Table 3.

**Table 3:** Associations of UCLA loneliness total score at baseline with FACT-BMT total score, days hospitalized, and immunological outcomes

		Association between UCLA loneliness total score at baseline (per each 5 unit increase) and the given outcome					
		Autologous transplant (*N* = 127)		Allogeneic transplant (*N* = 78)		All patients (*N* = 205)	
Outcome	Association measure	Estimate (95% CI)	P-value	Estimate (95% CI)	P-value	Estimate (95% CI)	P-value
FACT-BMT total score							
Day 100	Regression coefficient	-3.46 (-5.26, -1.65)	0.0003	-7.27 (-11.06, -3.48)	0.0004	-4.46 (-6.14, -2.78)	< 0.0001
1 year	Regression coefficient	-2.93 (-4.96, -0.91)	0.43	-5.74 (-11.50, 0.02)	0.051	-3.53 (-5.44, -1.62)	0.0004
Days hospitalized during transplant	Multiplicative effect on mean	1.03 (1.01, 1.06)	0.010	1.02 (0.98, 1.05)	0.33	1.02 (1.00, 1.05)	0.015
ANC (natural logarithm)							
Day 30	Regression coefficient	0.09 (0.04, 0.14)	0.001	-0.02 (-0.12, 0.07)	0.62	0.04 (-0.00, 0.09)	0.071
Day 100	Regression coefficient	0.07 (0.01, 0.13)	0.015	-0.07 (-0.18, 0.05)	0.25	0.02 (-0.04, 0.07)	0.52
1 year	Regression coefficient	0.00 (-0.08, 0.08)	0.97	-0.11 (-0.27, 0.04)	0.14	-0.03 (-0.10, 0.04)	0.41
ALC (natural logarithm)							
Day 30	Regression coefficient	0.00 (-0.05, 0.05)	0.93	-0.12 (-0.25, 0.00)	0.056	-0.05 (-0.10, 0.00)	0.068
Day 100	Regression coefficient	-0.00 (-0.05, 0.04)	0.90	-0.07 (-0.21, 0.06)	0.26	-0.03 (-0.08, 0.02)	0.25
1 year	Regression coefficient	-0.07 (-0.14, -0.00)	0.040	0.08 (-0.73, 0.24)	0.28	-0.03 (-0.10, 0.04)	0.39
AMC (natural logarithm)							
Day 30	Regression coefficient	0.03 (-0.02, 0.08)	0.23	-0.03 (-0.14, 0.08)	0.56	-0.00 (-0.05, 0.05)	0.96
Day 100	Regression coefficient	0.02 (-0.04, 0.07)	0.49	-0.09 (-0.24, 0.06)	0.25	-0.03 (-0.09, 0.03)	0.30
1 year	Regression coefficient	-0.04 (-0.09, 0.01)	0.14	-0.05 (-0.19, 0.08)	0.43	-0.04 (-0.09, 0.01)	0.15
ANC/AMC (natural logarithm)							
Day 30	Regression coefficient	0.06 (0.00, 0.12)	0.045	-0.01 (-0.14, 0.12)	0.83	0.03 (-0.02, 0.09)	0.23
Day 100	Regression coefficient	0.05 (-0.01, 0.11)	0.081	0.02 (-0.12, 0.16)	0.76	0.05 (-0.01, 0.11)	0.092
1 year	Regression coefficient	0.04 (-0.03, 0.11)	0.26	-0.06 (-0.15, 0.03)	0.20	0.01 (-0.05, 0.07)	0.72
ANC/ALC (natural logarithm)							
Day 30	Regression coefficient	0.09 (0.02, 0.16)	0.018	0.17 (0.03, 0.31)	0.015	0.12 (0.05, 0.18)	0.0005
Day 100	Regression coefficient	0.07 (0.00, 0.15)	0.048	0.01 (-0.15, 0.16)	0.92	0.05 (-0.02, 0.11)	0.16
1 year	Regression coefficient	0.08 (-0.02, 0.17)	0.10	-0.20 (-0.38, -0.02)	0.034	-0.00 (-0.09, 0.09)	> 0.99

CI = confidence interval. Regression coefficients, 95% CIs, and p-values result from multivariable linear regression models; regression coefficients are interpreted as the change in the mean outcome level per each 5 unit increase in UCLA loneliness total score. Multiplicative effects on the mean, 95% CIs, and p-values result from negative binomial regression models; multiplicative effects on the mean are interpreted as the multiplicative effect on the mean outcome level per each 5 unit increase in UCLA loneliness total score. Models were adjusted for age at transplant, sex, ethnicity, race, smoking history, current drinking, and BMI. Additionally, transplant subgroup was also adjusted for in analysis of all patients. P-values < 0.0071 were considered as statistically significant after applying a Bonferroni correction for multiple testing for the 7 general outcome measures that were assessed

**Fig. 2:**
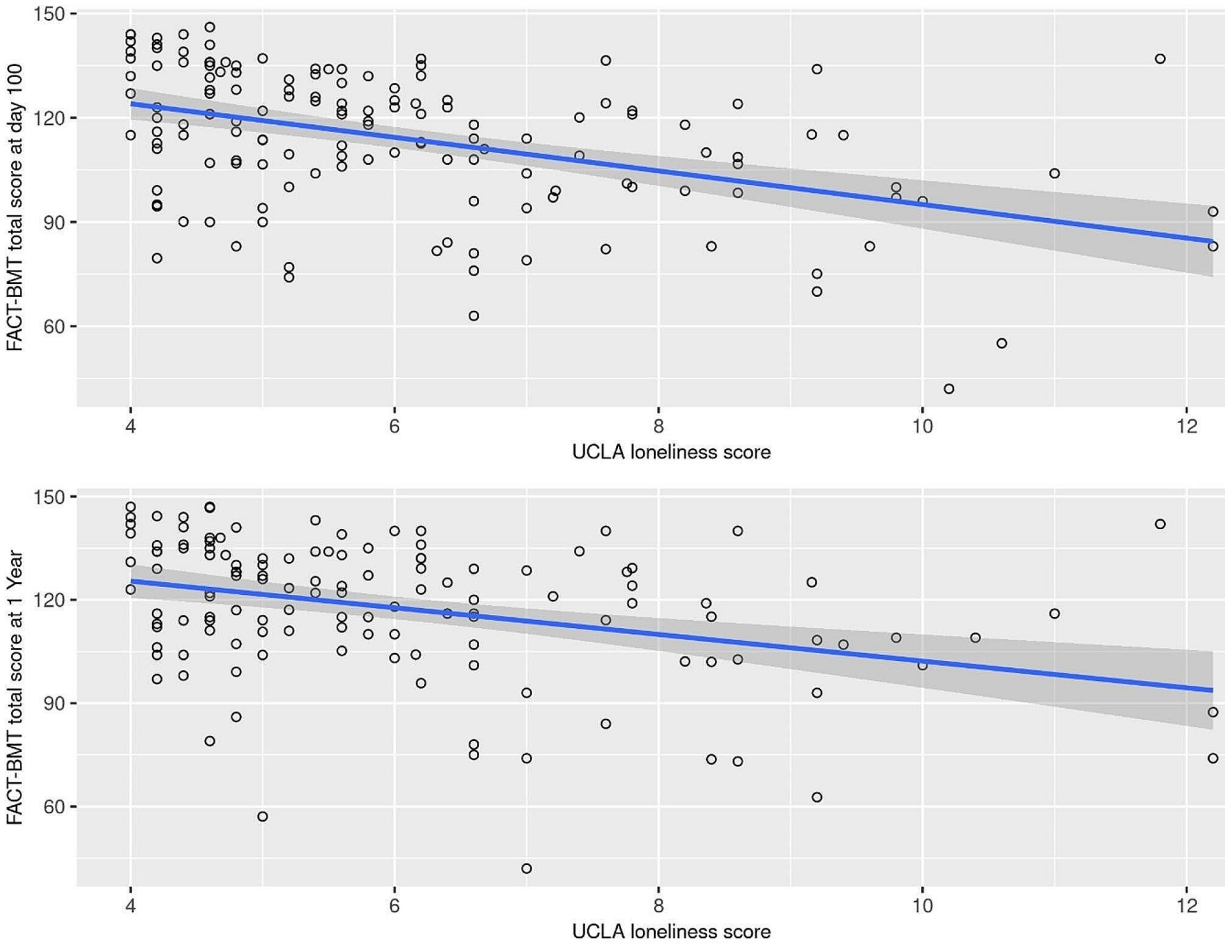
UCLA loneliness total score at baseline and FACT-BMT at Day 100 and 1 year in the overall group of 205 patients. The estimated regression line is displayed with 95% confidence intervals

Statistically significant (*p* < 0.0071) associations with a higher UCLA loneliness total score at baseline were noted for a lower FACT-BMT total score at day 100 in autologous transplants (*p* = 0.0003), allogeneic transplants (*p* = 0.0004), and the combined group (*p* < 0.0001, Fig. 2), and also for a lower FACT-BMT total score at 1 year in all patients (*p* = 0.0004, Fig. 2).

**Fig. 3:**
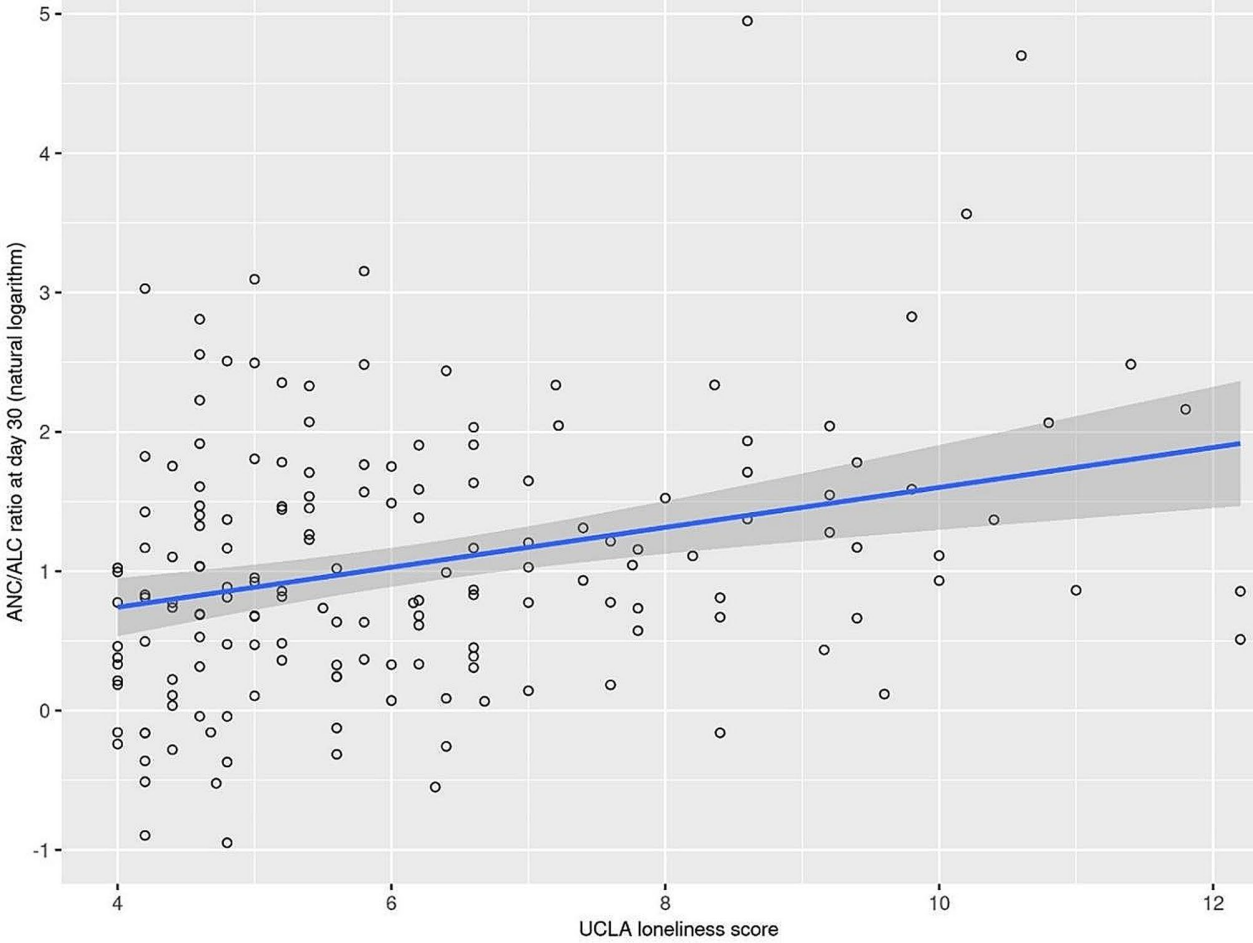
UCLA loneliness total score at baseline and ANC/ALC ratio at Day 30 in the overall group of 205 patients. The estimated regression line is displayed with 95% confidence intervals

Greater loneliness at baseline also predicted higher ANC/ALC ratios at Day 30 in the overall group (*p* = 0.0005, Fig. 3), with similar but only nominally significant (*p* < 0.05) findings observed for the separate autologous (*p* = 0.018) and allogeneic (*p* = 0.015) subgroups.

Interestingly, UCLA loneliness total score at baseline was not significantly associated with ANC/ALC ratio in the overall group at Day 100 (*p* = 0.16) or 1 year (*p* > 0.99), with a similar lack of a consistent association observed in the autologous and allogeneic subgroups (Table 3). Additionally, greater baseline loneliness was significantly associated with higher ANC values at Day 30 (*p* = 0.001). There were no other significant associations between UCLA loneliness total score and any of the other outcomes considered, though a number of nominally significant findings were observed (Table 3).

**Fig. 4:**
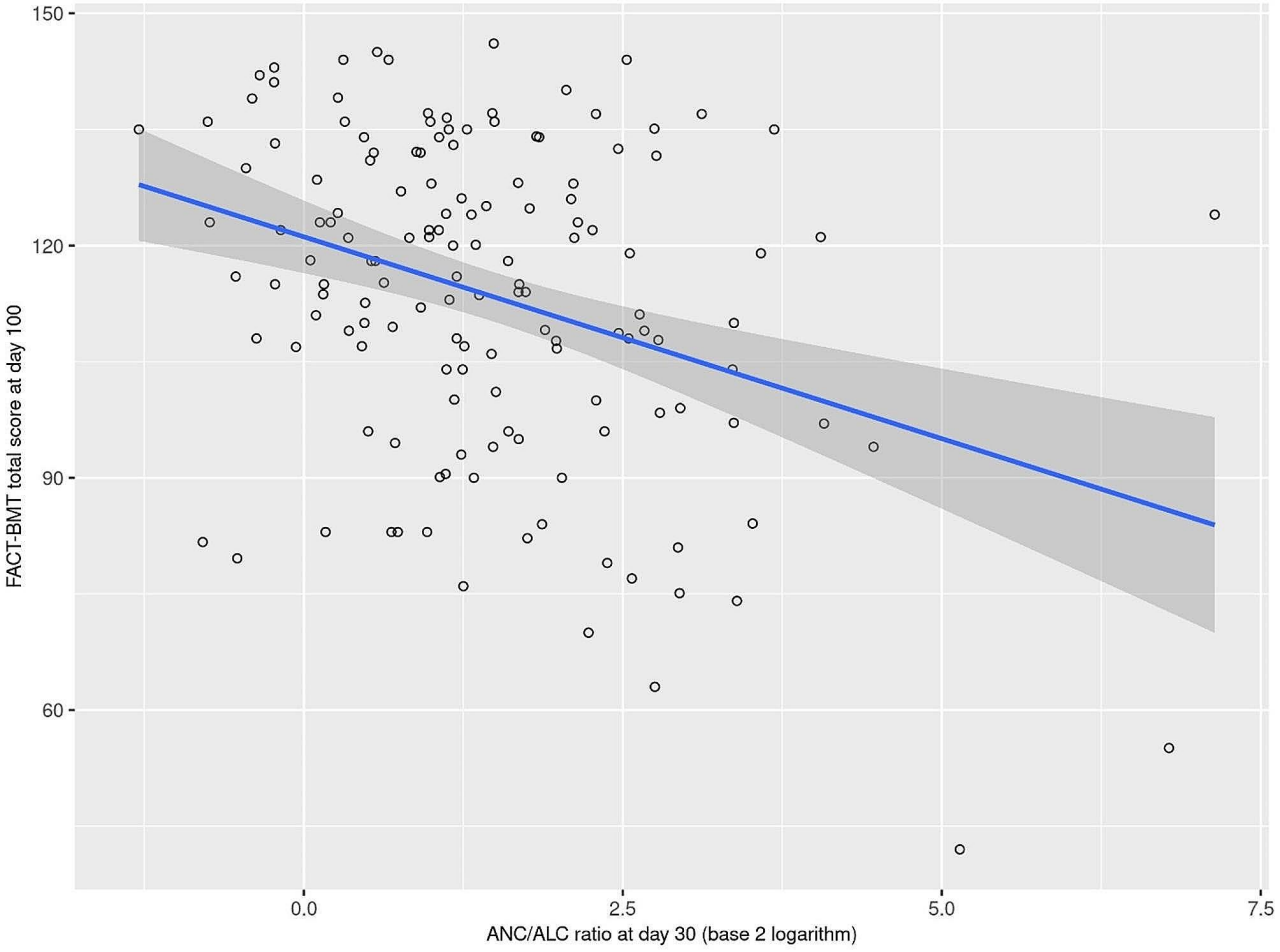
ANC/ALC ratio at Day 30 and FACT-BMT total score at Day 100 in the overall group of 205 patients. The estimated regression line is displayed with 95% confidence intervals

Additional analyses explored the association of ANC/ALC at Day 30 with HRQOL, as this ratio was significantly (*p* < 0.0071) associated with loneliness. As shown in Table 4, a higher ANC/ALC ratio at day 30 was associated with FACT-BMT total score at day 100 for the entire sample (*p* = 0.0001, Fig. 4) and allogenic transplant subgroup (*p* = 0.004), with a similar but non-significant trend noted for autologous transplants (*p* = 0.074). These associations with day 30 ANC/ALC ratio were not observed when examining FACT-BMT total score at 1-year follow-up (all *p* ≥ 0.40). Subsequently, we assessed the association between UCLA loneliness total score at baseline and FACT-BMT total score at day 100 and 1 year when additionally adjusting our multivariable models for ANC/ALC at day 30, in order to evaluate whether ANC/ALC at day 30 mediates the aforementioned associations (Supplemental Table 1). With this additional model adjustment, we still observed significant associations between baseline UCLA loneliness score and a lower FACT-BMT score at day 100 and 1 year, with very similar observed regression coefficients, indicating that ANC/ALC at day 30 does not mediate these associations.

Of interest, associations of ANC/ALC and ANC/AMC with UCLA loneliness total score and FACT-BMT total score measured at the same time point are examined in Supplemental Table 2. A significant association between greater ANC/ALC at baseline and UCLA loneliness total score at baseline was observed in the overall group (*p* = 0.003). Also of interest, comparisons of ANC, ALC, AMC, ANC/AMC, ANC/ALC, and FACT-BMT between different time points are shown in Supplemental Table 3.

**Table 4:** Associations of ANC/ALC ratio at Day 30 with FACT-BMT total score at Day 100 and 1 year

		Autologous transplant (*N* = 127)		Allogeneic transplant (*N* = 78)		All patients (*N* = 205)	
Outcome	Association measure	Estimate (95% CI)	P-value	Estimate (95% CI)	P-value	Estimate (95% CI)	P-value
Association of ANC/ALC at day 30 with:							
FACT-BMT total score							
Day 100	Regression coefficient	-3.63 (-7.62, 0.37)	0.074	-7.33 (-12.13, -2.54)	0.004	-5.84 (-8.34, -3.33)	0.0001
1 year	Regression coefficient	-1.33 (-5.74, 3.09)	0.55	2.74 (-3.81, 9.28)	0.40	-0.87 (-3.92, 2.17)	0.57

CI = confidence interval. Regression coefficients, 95% CIs, and p-values result from linear regression models; regression coefficients are interpreted as the change in the mean FACT-BMT total score per each doubling in ANC/ALC (which was considered on the base 2 logarithm scale). Models were adjusted for age at transplant, sex, ethnicity, race, smoking history, current drinking, and BMI. Additionally, transplant subgroup was also adjusted for in analysis of all patients

## Discussion

Findings support empirical and theoretical predictions [1, 12, 18] that the stress from loneliness impacts HRQOL and the immune system by increasing circulating neutrophil cells relative to lymphocytes [29, 31, 38, 39]. Specifically, higher pre-transplant loneliness predicted lower HRQOL 100 days post-transplant across the entire sample and transplant type (allogenic vs. autologous) and at 1 year for all patients. Additionally, elevated Day 30 ANC/ALC was found for patients experiencing higher levels of loneliness at baseline. Higher Day 30 ANC/ALC ratios suggest an imbalance of these innate-to-adaptive leukocytes in patients experiencing greater loneliness prior to HSCT. Elevated Day 30 ANC/ALC ratios in turn predicted HRQOL at 100 days post-transplant.

Loneliness at baseline appeared to have phasic associations with natural-to-adaptive immune recovery, with these Day 30 ANC/ALC pattern differences insignificant by Day 100 and 1-year post-HSCT. However, even though the impacts were mitigated by Day 100, Day 30 ANC/ALC trends were associated with poorer HRQOL 100 days following the transplant. Specifically, higher Day 30 ANC/ALC ratios independently predicted poorer overall HRQOL at day 100, with the association no longer significant at 1 year. Our findings indicate that Day 30 ANC/ALC ratio did not mediate the association between baseline loneliness and day 100 HRQOL, and therefore this association was observed across all levels of Day 30 ANC/ALC ratio. The ANC/ALC ratios has been identified as an indicator for inflammation and is considered a risk factor for several diseases [40], with high ANC/ALC predictive of shorter progression-free survival in HSCT patients [41]. Therefore, the phasic association of Day 30 ANC/ALC ratio with Day 100 HRQOL may be due to time limited impacts from inflammatory immunological recovery related to loneliness.

Findings of the current study are compelling because they provide support for loneliness theory [18, 21, 22], and biobehavioral models created specifically for HSCT patients [1, 12]. Theoretical predictions [18] that the unsafe feelings of loneliness cause stress, with humoral immune system consequences, are supported by the elevated ANC/ALC ratios in patients who experienced higher levels of loneliness prior to HSCT hospitalization. Immunological implications are of particular importance to HSCT patients, and it has been proposed that the critical period of early recovery is a time in which psychosocial factors may play a large role in outcomes [7, 12]. Findings support this contention, with natural-to-adaptive immunity recovery patterns associated with elevated loneliness predicting poorer HRQOL outcomes 100 Days after the transplant.

As noted by Knight and colleagues [1], there is a dearth of psychoneuroimmunological research on the HSCT population and this type of research is needed to identify pathways by which psychosocial factors impact outcomes. This study provides some evidence to address this need in the literature. However, findings should be considered within the confines of an individual study and more research is needed to further substantiate and explore the interplay of loneliness and other psychosocial factors on HSCT and other oncology populations. Limitations of our study include a moderate sample size, which may have contributed to a lack of findings for some immunological indices due to lack of power, and gaps in time interval measurement of HRQOL (Day 100, 1-year) may have missed intraindividual heterogeneity between these time points. Another consideration is that HSCT involves a complex interplay of several medical and other factors, which could have accounted for outcomes in the study; however, several demographic and medical factors were controlled for as necessary to reduce this threat. Study results should be interpreted with the understanding that the sample about a third of patients undergoing HSCT at Mayo Clinic Jacksonville during the study period as patients volunteered without compensation. Furthermore, immunological measures did not include data on flow cytometric assessment of lymphocyte subpopulations and future research should consider this measure, especially for allogeneic HSCT patients. A strength of the study is that it is prospective in design with loneliness measured pre-transplant. However, given the phasic responses in immune recovery patterns and HRQOL of life outcomes revealed in this study, future research should measure loneliness in a repeated measure format to better account for the role of loneliness in leukocyte recovery after a HSCT.

## Conclusion

This project is the first known study to prospectively investigate loneliness and immunological recovery in the HSCT population. The current study revealed that loneliness prior to HSCT is associated with ANC/ALC a month later, and that these distinct innate leukocyte system patterns predict degraded QOL and symptom control at 100 days post-transplant. Although causal connections cannot be determined in the current study, the results indicate that healthcare providers may have an opportunity to improve HSCT recovery by attending to patients’ perceptions of loneliness. Loneliness interventions show promise in reducing the distressing experience of social isolation [42] and may be effective in enhancing HSCT recovery.

### Electronic supplementary material

Below is the link to the electronic supplementary material.

**Supplementary Material 1:** Supplementary Tables 1–3

## Data Availability

The datasets used and/or analyzed during the current study are available from the corresponding author on reasonable request.

## Abbreviations

HSCT: hematopoietic stem cell transplantation
HRQOL: health-related quality of life
ANC: absolute neutrophil count
ALC: absolute lymphocyte count
AMC: absolute monocyte count
FACT-BMT: Functional Assessment of Cancer Therapy-Bone Marrow Transplant Scale
