# Supplementary material for: Loneliness, immunological recovery patterns, and health-related quality of life (HRQOL) outcomes in patients receiving hematopoietic stem cell transplantation
Source: BMC Psychol. 2024 Jan 19;12:40. doi: 10.1186/s40359-024-01535-w (PMC10797957; doi:10.1186/s40359-024-01535-w)
Supplement: Supplementary file 1 — Supplementary Material 1: Supplementary Tables 1–3 [file 40359_2024_1535_MOESM1_ESM.docx]

**Supplemental Table 1: Associations of UCLA loneliness total score with FACT-BMT total score at day 100 and 1 year when additionally adjusting multivariable models for ANC/ALC at day 30**

|  |  | Association between UCLA loneliness total score (per each 5 unit increase) and FACT-BMT total score | | | | | |
| --- | --- | --- | --- | --- | --- | --- | --- |
|  |  | Autologous transplant (N=127) | | Allogeneic transplant (N=78) | | All patients (N=205) | |
| Outcome | Association measure | Estimate (95% CI) | P-value | Estimate (95% CI) | P-value | Estimate (95% CI) | P-value |
| Original multivariable analysis |  |  |  |  |  |  |  |
|  |  |  |  |  |  |  |  |
| FACT-BMT total score |  |  |  |  |  |  |  |
| Day 100 | Regression coefficient | -3.46 (-5.26, -1.65) | 0.0003 | -7.27 (-11.06, -3.48) | 0.0004 | -4.46 (-6.14, -2.78) | <0.001 |
| 1 year | Regression coefficient | -2.93 (-4.96, -0.91) | 0.43 | -5.74 (-11.50, 0.02) | 0.051 | -3.53 (-5.44, -1.62) | 0.0004 |
|  |  |  |  |  |  |  |  |
| Additionally adjusting for ANC/ALC at day 30 |  |  |  |  |  |  |  |
|  |  |  |  |  |  |  |  |
| FACT-BMT total score |  |  |  |  |  |  |  |
| Day 100 | Regression coefficient | -3.40 (-5.43, -1.38) | 0.001 | -5.49 (-9.87, -1.11) | 0.016 | -3.90 (-5.78, -2.01) | <0.001 |
| 1 year | Regression coefficient | -3.34 (-5.62, -1.06) | 0.005 | -7.07 (-13.09, -1.06) | 0.023 | -4.15 (-6.25, -2.05) | 0.0002 |
| CI=confidence interval. Regression coefficients, 95% CIs, and p-values result from linear regression models; regression coefficients are interpreted as the change in the mean FACT-BMT total score per each 5 unit increase in UCLA loneliness total score. Original multivariable models were adjusted for age at transplant, sex, ethnicity, race, smoking history, current drinking, and BMI. Additionally, transplant subgroup was also adjusted for in analysis of all patients. | | | | | | | |

**Supplemental Table 2: Associations of ANC/ALC and ANC/AMC with UCLA loneliness total score and FACT-BMT total score when measured at the same time point**

| Association/Patient group | Regression coefficient (95% CI) | P-value |
| --- | --- | --- |
| Association between baseline ANC/ALC and baseline UCLA loneliness total score |  |  |
| Autologous transplant (N=127) | 0.23 (-0.03, 0.48) | 0.077 |
| Allogeneic transplant (N=78) | 0.28 (0.05, 0.51) | 0.017 |
| All patients (N=205) | 0.25 (0.08, 0.42) | 0.0034 |
| Association between baseline ANC/AMC and baseline UCLA loneliness total score |  |  |
| Autologous transplant (N=127) | 0.15 (-0.19, 0.49) | 0.38 |
| Allogeneic transplant (N=78) | -0.11 (-0.41, 0.19) | 0.48 |
| All patients (N=205) | 0.04 (-0.18, 0.27) | 0.72 |
| Association between day 100 ANC/ALC and day 100 FACT-BMT total score |  |  |
| Autologous transplant (N=127) | -2.62 (-6.91, 1.67) | 0.25 |
| Allogeneic transplant (N=78) | -1.01 (-7.94, 5.92) | 0.77 |
| All patients (N=205) | -1.96 (-5.38, 1.46) | 0.29 |
| Association between day 100 ANC/AMC and day 100 FACT-BMT total score |  |  |
| Autologous transplant (N=127) | -0.91 (-6.47, 4.65) | 0.85 |
| Allogeneic transplant (N=78) | -1.62 (-9.09, 5.84) | 0.65 |
| All patients (N=205) | -1.05 (-5.10, 3.00) | 0.70 |
| Association between 1 year ANC/ALC and 1 year FACT-BMT total score |  |  |
| Autologous transplant (N=127) | -3.36 (-7.83, 1.10) | 0.091 |
| Allogeneic transplant (N=78) | -4.93 (-13.82, 3.96) | 0.26 |
| All patients (N=205) | -3.44 (-7.46, 0.58) | 0.074 |
| Association between 1 year ANC/AMC and 1 year FACT-BMT total score |  |  |
| Autologous transplant (N=127) | -4.28 (-10.17, 1.62) | 0.14 |
| Allogeneic transplant (N=78) | -2.38 (-20.31, 15.55) | 0.79 |
| All patients (N=205) | -2.62 (-8.59, 3.35) | 0.39 |
| CI=confidence interval. Regression coefficients, 95% CIs, and p-values result from multivariable linear regression models; regression coefficients are interpreted as the change in the mean outcome UCLA total loneliness score or FACT-BMT total score per each doubling in ANC/ALC or ANC/AMC (both of which were considered on the base 2 logarithm scale). Models were adjusted for age at transplant, sex, ethnicity, race, smoking history, current drinking, and BMI. Additionally, transplant subgroup was also adjusted for in analysis of all patients. | | |

**Supplemental Table 3: Comparisons of ANC, ALC, AMC, ANC/AMC, ANC/ALC, and FACT-BMT between different time points**

| Time point comparison | ANC | ALC | AMC | ANC/AMC | ANC/ALC | FACT-BMT |
| --- | --- | --- | --- | --- | --- | --- |
| Baseline vs. 30 days |  |  |  |  |  |  |
| Mean difference (95% CI) | -1.65 (-2.73, -0.57) | -0.00 (-0.15, 0.14) | 0.32 (0.22, 0.42) | -4.95 (-7.78, -2.12) | -1.74 (-4.73, 1.24) | N/A |
| P-value | 0.0029 | 0.97 | <0.0001 | 0.0007 | 0.25 | N/A |
| Baseline vs. 100 days |  |  |  |  |  |  |
| Mean difference (95% CI) | -1.89 (-3.11, -0.66) | 0.04 (-0.14, 0.23) | -0.01 (-0.12, 0.10) | -2.92 (-5.75, -0.09) | -1.99 (-3.51, -0.47) | N/A |
| P-value | 0.0027 | 0.63 | 0.93 | 0.044 | 0.011 | N/A |
| Baseline vs. 1 year |  |  |  |  |  |  |
| Mean difference (95% CI) | -1.70 (-3.19, -0.20) | 0.25 (0.05, 0.46) | -0.02 (-0.11, 0.07) | -3.07 (-5.61, -0.53) | -2.57 (-4.44, -0.70) | N/A |
| P-value | 0.026 | 0.016 | 0.71 | 0.018 | 0.0074 | N/A |
| 30 days vs. 100 days |  |  |  |  |  |  |
| Mean difference (95% CI) | -0.41 (-0.84, 0.03) | 0.05 (-0.10, 0.21) | -0.38 (-0.47, -0.28) | 2.95 (1.45, 4.46) | -2.10 (-4.39, 0.19 | N/A |
| P-value | 0.069 | 0.52 | <0.0001 | 0.0002 | 0.072 | N/A |
| 30 days vs. 1 year |  |  |  |  |  |  |
| Mean difference (95% CI) | 0.51 (-0.39, 1.40) | 0.26 (0.06, 0.45) | -0.35 (-0.43, -0.27) | 2.65 (1.66, 3.63) | -2.50 (-5.58, 0.59) | N/A |
| P-value | 0.26 | 0.011 | <0.0001 | <0.0001 | 0.11 | N/A |
| 100 days vs. 1 year |  |  |  |  |  |  |
| Mean difference (95% CI) | 0.93 (0.05, 1.81) | 0.34 (0.17, 0.52) | 0.06 (0.01, 0.10) | 0.03 (-1.37, 1.42) | -0.18 (-0.87, 0.52) | 1.94 (-0.86, 4.74) |
| P-value | 0.038 | 0.0002 | 0.011 | 0.97 | 0.61 | 0.17 |
| P-values result from a paired t-test. | | | | | | |
